# Tuning Light Emission towards White Light from a Naphthalenediimide-Based Entangled Metal-Organic Framework by Mixing Aromatic Guest Molecules

**DOI:** 10.3390/polym10020188

**Published:** 2018-02-14

**Authors:** Rebeca Sola-Llano, Virginia Martínez-Martínez, Shuhei Furukawa, Yohei Takashima, Iñigo López-Arbeloa

**Affiliations:** 1 Departamento de Química Física, Universidad del País Vasco, UPV/EHU, Apartado 644, 48080 Bilbao, Spain; rebeca.sola@ehu.eus (R.S.-L.); inigo.lopezarbeloa@ehu.eus (I.L.-A.); 2 Institute for Integrated Cell-Material Sciences (WPI-iCeMS), Kyoto University, Yoshida, Kyoto 606-8501, Japan; shuhei.furukawa@icems.kyoto-u.ac.jp; 3 Department of Nanobiochemistry, Frontiers of Innovative Research in Science and Technology (FIRST), Konan University, 7-1-20 Minatojimaminamimachi, Chuo-ku, Kobe 650-0047, Japan; takashim@center.konan-u.ac.jp

**Keywords:** entangled MOF (metal organic framework), aryl guests, charge transfer, white light emission, CIE (Comission Internationale de l´Éclairage) coordinates, UV illumination, white light emission

## Abstract

Taking advantage of the outstanding properties of a naphthalenediimide-based entangled porous coordination polymer, a simple strategy for the achievement of white light emission is herein presented. The dynamic structural transformation of the [Zn_2_(bdc)_2_(dpNDI)]*_n_* metal-organic framework enhances the interactions with aryl-guests giving rise to different luminescence colors upon UV (ultraviolet) illumination. Thus, through the rational selection of those small aromatic guest molecules with different electron donor substituents at the appropriate proportion, the emission color was tuned by mixture ratio of guest molecules and even white light emission was achieved. Furthermore, domains in large crystals with a complementary response to linearly polarized light were noticed.

## 1. Introduction

White light emitters are of extreme significance for lighting and display systems [1,2,3], and necessary in any technological device used in everyday life. Indeed, wide research has been performed on this topic over decades [3,4,5]. As a result of many investigations, there are plenty of strategies applied to generate white light. Those strategies vary from the combinations of different semiconductor quantum dots (QD) to newly-formulated organic-inorganic fluorescent (nano) composites [6,7].

In this context, metal-organic frameworks (MOFs), also called porous coordination polymers (PCPs), arise as hugely promising materials due to their multiple possibilities. Indeed, these MOFs are built up by strong coordination bonds between transition-metal cations (or clusters) and multidentate organic linkers, and considering their hybrid composition, they exhibit advantages of both organic and inorganic components. Depending on the linker, the metal and the coordination geometry adopted, they offer a wide variety of highly periodical structures, with intrinsic porosity and straightforward surface decoration [8,9]. Owing to this versatility, throughout the rational choice of the organic linker, MOFs have found usefulness in numerous applications, such as gas separation, storage and purification, catalysis, molecular sensing as well as in biomedicine and photonic technologies [10,11,12,13,14,15].

Furthermore, the research on MOFs is currently expanding towards new applications wherein their porous nature and physical properties are synergistically linked. In particular, MOFs appear as fascinating platforms for the development of luminescent materials due to their potential collaborative multifunctionalities. The luminescence properties in MOFs can be induced by different sources, such as the organic linkers (π-conjugated ligands), the coordinated metal ions (lanthanides) or antenna effects between them (ligand-to-metal charge transfer “LMCT” or metal-to-ligand charge transfer “MLCT” processes) [16,17,18,19,20]. In this context, the combination of some luminescent components in the scaffold has led to white light emission, for example, linking blue-emitting organic ligands to different lanthanide metals with characteristic emission bands in the green and red region of the electromagnetic spectrum, being simultaneously part of the structure of the MOF [21,22].

Besides the inherent luminescent properties of some MOFs, the capability to accommodate guest species in their well-defined porous structure also leads to very attractive luminescent features, denoted as guest-induced luminescence. Thus, by the confinement of guests into the nanopores, self-quenching processes even at high concentrations can be avoided, increasing the emission capacity of the guests. On the other hand, the fluorescence can also be enhanced by the tight encapsulation of guests into MOFs with rigid structures matching the molecular dimensions to the pore size [18,23,24]. As a recent example, the confinement of dyes with emission bands in different ranges of the electromagnetic spectrum in the right proportion into a rigid MOF, resulted in a white light emitting material [3].

Nevertheless, there are many other ways based on guest-induced luminescence to attain MOF-based materials with interesting emissive properties. For instance, charge transfer complexes or exciplexes (in the excited state) can be formed between the organic linkers and guest species leading to new species or new states with characteristic fluorescent bands different from those of the isolated host and guest species. There can also exist energy transfer processes between host frameworks and guest species that can result in the shift of emission wavelength, luminescence quenching or antenna effect, or even structural transformations due to guest accommodation that result in differences in the luminescent intensity [16,25].

Some MOFs show unique properties as they can form entangled structures, such as the aforementioned possibility of undergoing structural transformations [26,27]. In spite of the disadvantage of having reduced the void spaces, the entanglement of chemically noninterconnected frameworks offers dynamic structures by a dislocation of their mutual positions. This flexibility though spaces upon guest-exchange, denoted as dynamic confinement, is a consequence of specific host–guest interactions. This structural property can trigger unique luminescent properties not observed in other hybrid systems.

**Figure 1:**
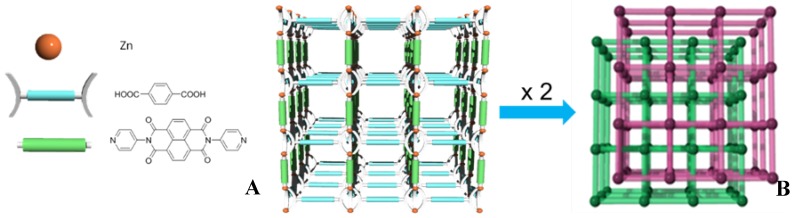
(**A**) Illustrative representation of one of the two identical frameworks that compose the [Zn_2_(bdc)_2_(dpNDI)]*_n_* MOF indicating its different components; (**B**) an schematic representation of the interpenetration of the two identical frameworks.

Particularly in this work, the MOF formulated as [Zn_2_(bdc)_2_(dpNDI)]*_n_*, with *N*,*N*′-dipyrid-4-yl-1,4,5,8-naphthalenediimide (dpNDI) incorporated as photoactive pillar, consists of two identical intergrown frameworks, forming an entangled structure (Figure 1).

**Figure 2:**
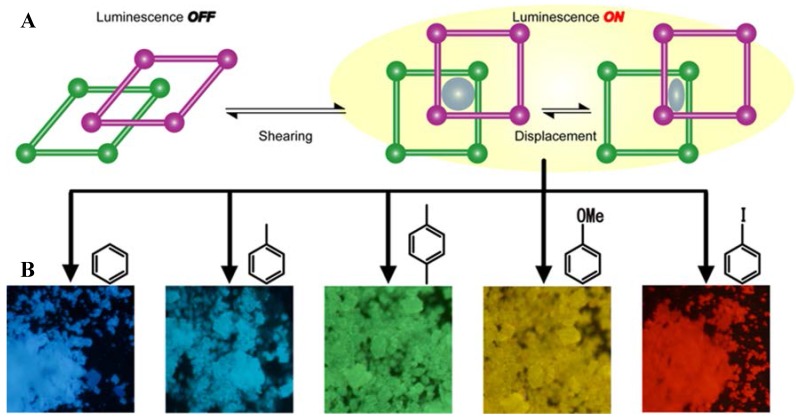
**(A**) Structural dynamics of the MOF on the accommodation of different guest molecules. (**B**) fluorescence image of [Zn_2_(bdc)_2_(dpNDI)]*_n_* powder with different VOC (Volatile Organic Compounds)molecules occluded in the pore: benzene, toluene, *p*-xylene, anisole and iodobenzene.

This MOF suffers a structural transformation by the incorporation of aryl guest molecules in its pores as a consequence of specific host-guest interactions (Figure 2). This dynamic guest confinement on the PCP has demonstrated a highly selective molecule sensing able to decode small aromatic guests by the change in the luminescence color visualized by the naked eye under UV illumination [28,29]. The final host-guest system can render different luminescence colors from blue to cyan, green, yellow or red, because of the confinement of aryl guest species with different substitution pattern into the pores of this photoactive MOF. The different emission colors visualized are the result of the formation of different charge transfer (CT) complexes, which depends mainly on the ionization potential of the aryl guest molecules [28,29]. 

By taking advantage of the broad color palette that can be easily covered with this versatile hybrid system due to the guest-dependent emission, white color emission is now pursued with this [Zn_2_(bdc)_2_(dpNDI)]*_n_* as host scaffold with the simultaneous incorporation of some of those small aromatic molecules into the pores in an adequate proportion. White light emission is to be envisaged not only in [Zn_2_(bdc)_2_(dpNDI)]*_n_* crystalline powders but also in big single crystals.

## 2. Materials and Methods

### 2.1. Synthesis

{[Zn_2_(bdc)_2_(dpNDI)]·4(DMF)}*_n_* is prepared according to the previous literature [30]. Briefly, a mixture containing Zn(NO_3_)_2_·6H_2_O, H_2_bdc and dpNDI was suspended in *N,N*-dimethyl-formamide (DMF) and heated to 95 °C for a period of 3 days. The slightly yellow crystals were then harvested.

### 2.2. Characterization

The fluorescent and phosphorescent quantum yields were measured using an absolute photoluminescence quantum yield measurement system equipped with an integrating sphere (C9920-02, Hamamatsu, Japan). Fluorescence color images were recorded with an optical inverted microscope with epi configuration (BX51, Olympus, London, UK) equipped with a color CCD (DP72, Olympus). Samples were excited with UV light by respective Chroma band-pass filters (350/50), and emission was collected with a Chroma cutoff filter (Bellows Falls, VT, USA) from 400 nm. Fluorescence spectra of the particles (powder or single crystals) were recorded by a fiber coupling from Olympus to an Edinburgh Instruments spectrofluorimeter (model FLSP 920, Edinburgh, UK). 

Polarization fluorescence single-particle measurements were performed in a time-resolved fluorescence confocal microscope (model Micro Time 200, PicoQuant, Berlin, Germany). The excitation was performed at 410 nm with a picosecond pulsed diode laser with 100 ps pulses at 5 MHz repetition rate. The fluorescence signal was collected by the same objective and focused (via a 50 μm pinhole) onto avalanche photodiode detectors (Micro-Photon-Devices, PDM, Bolzano, Italy). Polarization measurements were performed with unpolarized excitation light, and then the emission signal collected was divided by a polarizer beam splitter (Bellows Falls, VT, USA) into two mutually perpendicular polarization orientation beams, which are simultaneously detected by two detector channels. We analyzed the dichroic ratio (*D* = *I*_∥_/*I*_⊥_), defined as the relation between the emission intensity counts collected for two perpendicularly polarized radiations (parallel to the main c-axis of the crystal over perpendicular to it).

## 3. Results and Discussion

**Figure 3:**
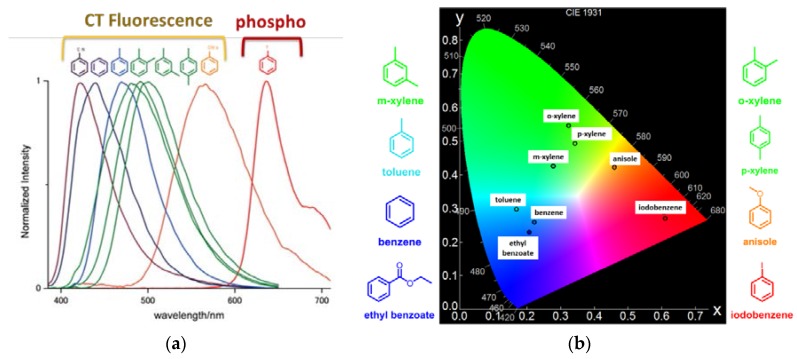
(**a**) Height-normalized emission spectra (excitation at 370 nm) and (**b**) CIE coordinates of PCP with different aromatic guests.

As previously explained, the key factor for the appearance of luminescence in this hybrid system is the high electron acceptor capability of the NDI compound [31,32,33], which is able to form charge transfer (CT) complexes with aryl-derivatives as electron donors with different substitution. These CT complexes are characterized by new red-shifted emission bands with respect to naphthalenediimide (NDI) ligand [34], as it has been shown in aromatic hydrocarbon solvents [35]. Importantly, in the present case those interactions between the aryl guests, such as benzene, toluene, xylene and anisole (Figure 2) and the NDI ligand are maximized by structural dynamics in response to guest accommodation. In this sense, the higher the ionization potential of the aromatic guest species, the larger the bathochromic shift of the emission band is obtained (Figure 3a), with quantum yields up to ten times higher than that of the mixture of the NDI derivatives and the aromatic compounds in solution [29,34]. Note here that, not only the luminescence properties are based on CT complexation, but for the occlusion of halogenated benzene derivative (for example, iodobenzene) phosphorescence emission at room temperature and in aerated medium is turned on (Figure 3a) in the entangled MOF. This emission is characteristic by a red emission at around 640 nm with a lifetime in the order of 100 μs after excitation at 370 nm (in the UV region) [36].

The first step before selecting the guest-combinations and their proportion into the MOF to trigger white light was to determine the CIE coordinates of the emission of selective guests occluded separately into the PCP. Figure 3b shows the results obtained for ethyl benzoate, benzene, toluene, ortho-xylene, meta-xylene, para-xylene, anisole and iodobenzene molecules once incorporated within the MOF pores.

Now, different mixtures of guests were then chosen to promote white light emission from the MOF. First, a combination of ethyl benzoate (blue), para-xylene (green) and iodobenzene (red) was chosen to cover the emission of the three primary colors. Note here that among the guests that activate the emission in the green region of the visible spectra, para-xylene was selected since it promotes the highest fluorescence quantum yield with respect to the other xylene species [29]. A second combination was attempted by mixing toluene (cyan), anisole (orange) and iodobenzene (red) into the MOF. Iodobenzene was chosen in both cases since it is the only aromatic component that triggers pure red emission.

**Figure 4:**
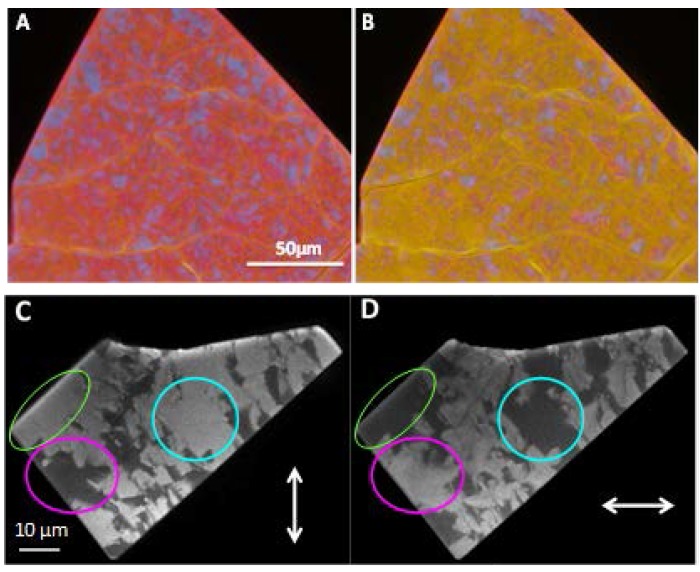
Fluorescence image of a MOF big crystal previously immersed in toluene and iodobenzene, (**A**) just after the addition of anisole, and (**B**) 10 min later. (**C**,**D**) Polarization images of a PCP crystal immersed in toluene under 410 nm excitation. White arrows indicate the direction of the polarizers. Circles highlight domains with complementary response to linear polarized light.

In all the cases, the small aromatic molecules were embedded into the pores as following; mixtures of the organic components in different ratios were firstly prepared, and then MOF solids (powder or crystals) were added to, followed by keeping them in the liquid mixture for at least an hour to obtain a homogeneous distribution of the organic guests in the materials. In some cases, the longer immersion time was required for big single crystals to ensure an adequate diffusion of the guests along the pores. For a better understanding of this diffusion phenomenon, Figure 4 shows a big crystal that was previously immersed in a toluene (cyan)/iodobenzene (red) mixture in a 1:1 ratio and then anisole (yellow) was added dropwise under the microscope. Figure 4A shows a fluorescence image of the crystal just after the addition of the anisole, and Figure 4B the same after 10 min. In both images, different emission domains are clearly visualized across the single crystal. In the first steps of the anisole diffusion, only the borders of the domains appear colored in yellow (Figure 4A). Then, its characteristic yellow fluorescence is spread practically over the whole particle as the diffusion process goes on (Figure 4B). Therefore, in order to obtain white light emission in big crystals the best strategy is to immerse them into the desired mixtures of aromatic guests for several hours, leading to a homogeneous guest distribution in a whole crystal.

The domains noticed in the MOF crystals show a complementary response to the linearly polarized light (Figure 4C,D). Discrete areas (>1 µm) switch on or off depending on the direction of light-polarization, indicating the existence of macroscopic twinned crystals, as also demonstrated with the crystallographic analysis [29]. Indeed, by polarized fluorescence microscopy it is easy to recognize the distribution, size and shape of the different twin domains. The detection of those twins would help the structural determination since microscopic twinning cannot be detected without prior suspicion.

Finally, white light emission was achieved in both MOF powders and single crystals with different guest combinations. The optimum proportion for each combination and the CIE coordinates for the corresponding emission are summarized in Table 1.

**Figure 5:**
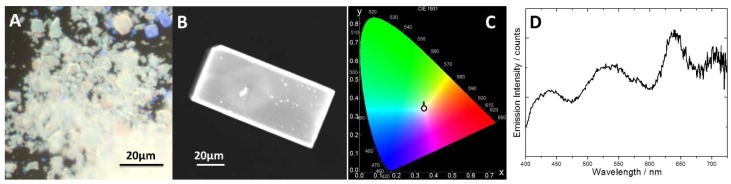
Fluorescence images of (**A**) powder and (**B**) single crystal MOF immersed in toluene/anisole/iodobenzene mixture in 7:4:2 proportion under UV excitation light (325–375 nm band pass filter). (**C**) CIE diagram for the single crystal shown in (**B**,**D**). Emission spectrum recorded under UV illumination (325–375 nm band pass filter) of the MOF powder immersed in ethyl benzoate, p-xylene and iodobenzene in a 5:1:2.6 proportion.

For the first combination of organic guests (ethyl benzoate: p-xylene: iodobenzene), the proportion of the different guests for white light emission in powders or in single crystals was slightly different to each other. In the first case, white light was generated with a solvent proportion of 5:1:2.6, obtaining CIE coordinates of 0.34, 0.33 (x, y); while in the case of single crystals a proportion of 2:1:1 was needed for CIE coordinates of 0.33, 0.35 (x, y). This difference is most likely attributed to a more impeded diffusion along the pores of the bulkier p-xylene (with two substituent groups, Figure 3) with respect to the other guests in big single crystals. On the other hand, for toluene: anisole: iodobenzene mixture, the optimum guest proportion to obtain white light emission was 7:4:2 for both powders and single crystals, with CIE coordinates of 0.35, 0.34 (x, y) (Figure 5).

It has been revealed that independently of the MOF morphology (big crystals or powder), white light can be produced by embedding three aromatic species into the pores of [Zn_2_(bdc)_2_(dpNDI)]*_n_*. The emission spectra recorded in all cases under ultraviolet light illumination show that the luminescence of the samples covers the whole visible spectrum (Figure 5D), with similar emission intensities in the whole range. All the cases mentioned above showed a fluorescence quantum yield of around 6–8% (ϕ_fl_: 0.06–0.08). Note here that this emission efficiency was far to be found in solution (ϕ_fl_ ≤ 0.01) [34], since the CT interaction is only maximized by the confinement into the MOF pores.

Hence, different combination of guest mixtures can be used for this purpose, and many others could also lead to white light emission. Here, only the mixtures that led the highest emission efficiencies have been presented. Note that in the study presented herein white light emission has been pursued as an example of emission color; furthermore, due to the versatility of the emission-range of the MOF depending on the guest molecules, any desired emission color can be obtained.

## 4. Conclusions

[Zn_2_(bdc)_2_(dpNDI)]*_n_* hybrid structure provides an easy and straightforward method to obtain different emission colors in the whole visible spectrum under ultraviolet excitation light. By embedding diverse, small aromatic guest molecules, the whole visible spectral range can be covered. Furthermore, through the proper combination of different guests and simply by immersing the MOF powders or crystals into the mixture of compounds, white light emission can be attained.

## Figures and Tables

**Table 1:** VOC proportions for the achievement of the white light with the different mixtures and their corresponding CIE coordinates, both in powder sample and single crystals.

Mixture	Proportion		CIE Coordinates (x, y)	
	Powder	Crystals	Powder	Crystals
ethyl benzoate: p-xylene: iodobenzene	5:1:2.6	2:1:1	0.34, 0.33	0.33, 0.35
toluene: anisole: iodobenzene	7:4:2	7:4:2	0.35, 0.34	0.35, 0.34
